# Supplementary material for: A dual mechanism promotes switching of the Stormorken STIM1 R304W mutant into the activated state
Source: Nat Commun. 2018 Feb 26;9:825. doi: 10.1038/s41467-018-03062-w (PMC5827659; doi:10.1038/s41467-018-03062-w)
Supplement: Supplementary file 1 — Supplementary Information [file 41467_2018_3062_MOESM1_ESM.pdf]

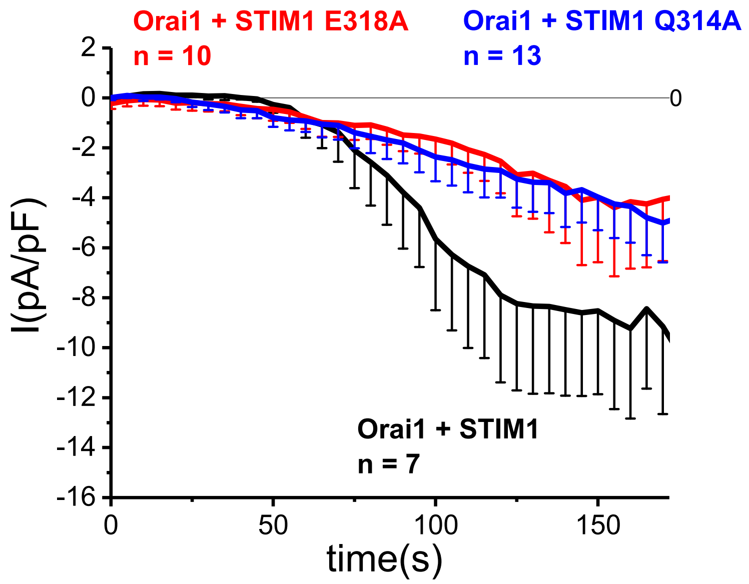

Supplementary Figure 1. Patch clamp experiments of HEK293 cells co-expressing YFP-Orai1 and CFP-STIM1 (black, n=7), YFP-Orai1 and CFP-STIM1 E318A (red, n=10) or YFP-Orai1 and CFP-STIM1 Q314A (blue, n=13), respectively. Error bars are defined as s.e.m.

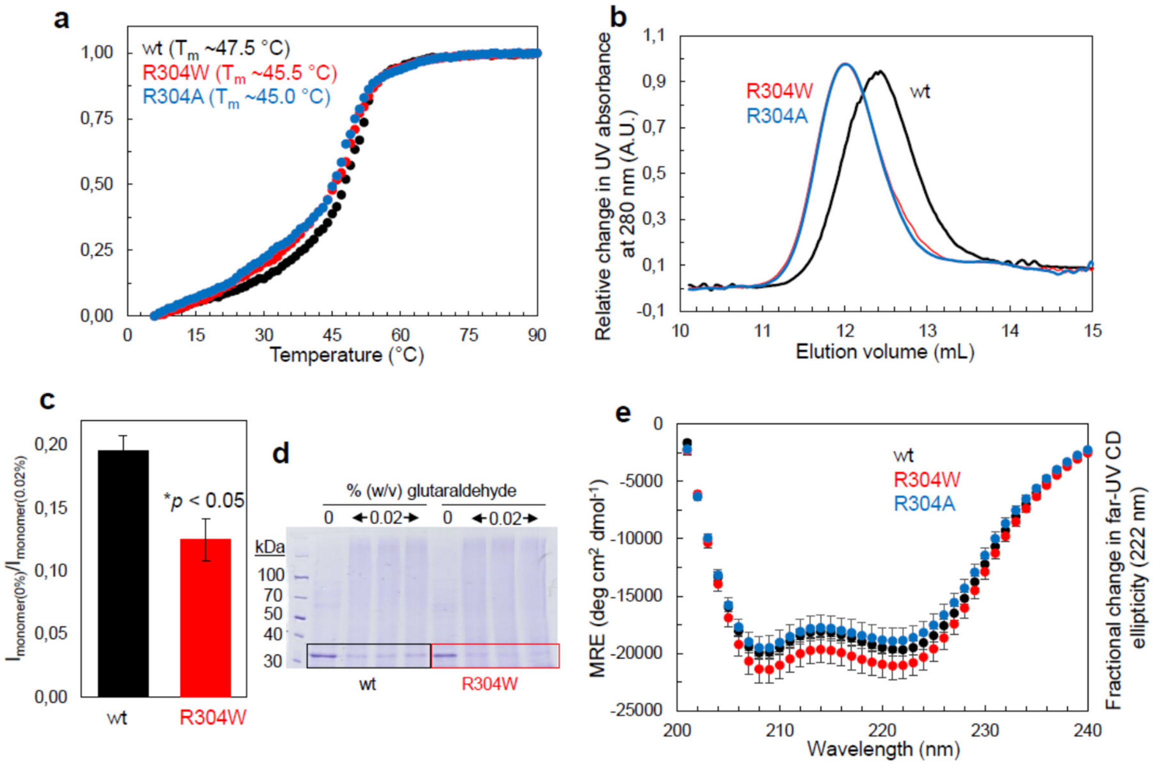

Supplementary Figure 2. Biochemical and biophysical analysis of recombinant wt and R304 mutant OASFext. (a) Thermal stability of OASFext proteins. The R304A and R304W mutant proteins showed apparent Tm values which were ~2 °C less than the wt protein. (b) SEC analysis of OASFext proteins. The R304A and R304W mutant proteins exhibited earlier elution volumes, suggesting a relatively more extended conformation compared to the wt OASFext protein. The identity of the protein and intact nature was confirmed by Coomassie blue stained 15% (w/v) SDS-PAGE gels. (c) Glutaraldehyde crosslinking assessment of OASFext proteins. The R304W protein showed significantly lower persistence of monomer in the presence of the crosslinking reagent compared to wt protein, suggesting a higher propensity for self-association. Statistics are Student's t-test. (d) Representative Coomassie blue stained 15% (w/v) SDS-PAGE gel assessing protein crosslinking. The boxes bound the OASFext monomer bands in each case, where the intensity of the monomer bands for the R304W protein is suppressed in the presence of glutaraldehyde. (e) Far-UV CD spectra of OASFext proteins. The two minima at ~208 and 222 nm are indicative of high α-helicity. The trend for enhanced negative ellipticity observed for R304W is consistent with increased α-helicity. In (*a*) – (*e*) wt, R304W, R304A data are black, red and blue, respectively. Statistics are Student's t-test. Data in (*c*) and (*e*) are means ± s.e.m. of n=3 experiments. Data in (*a*) and (*d*) are representative of two and three experiments, respectively.

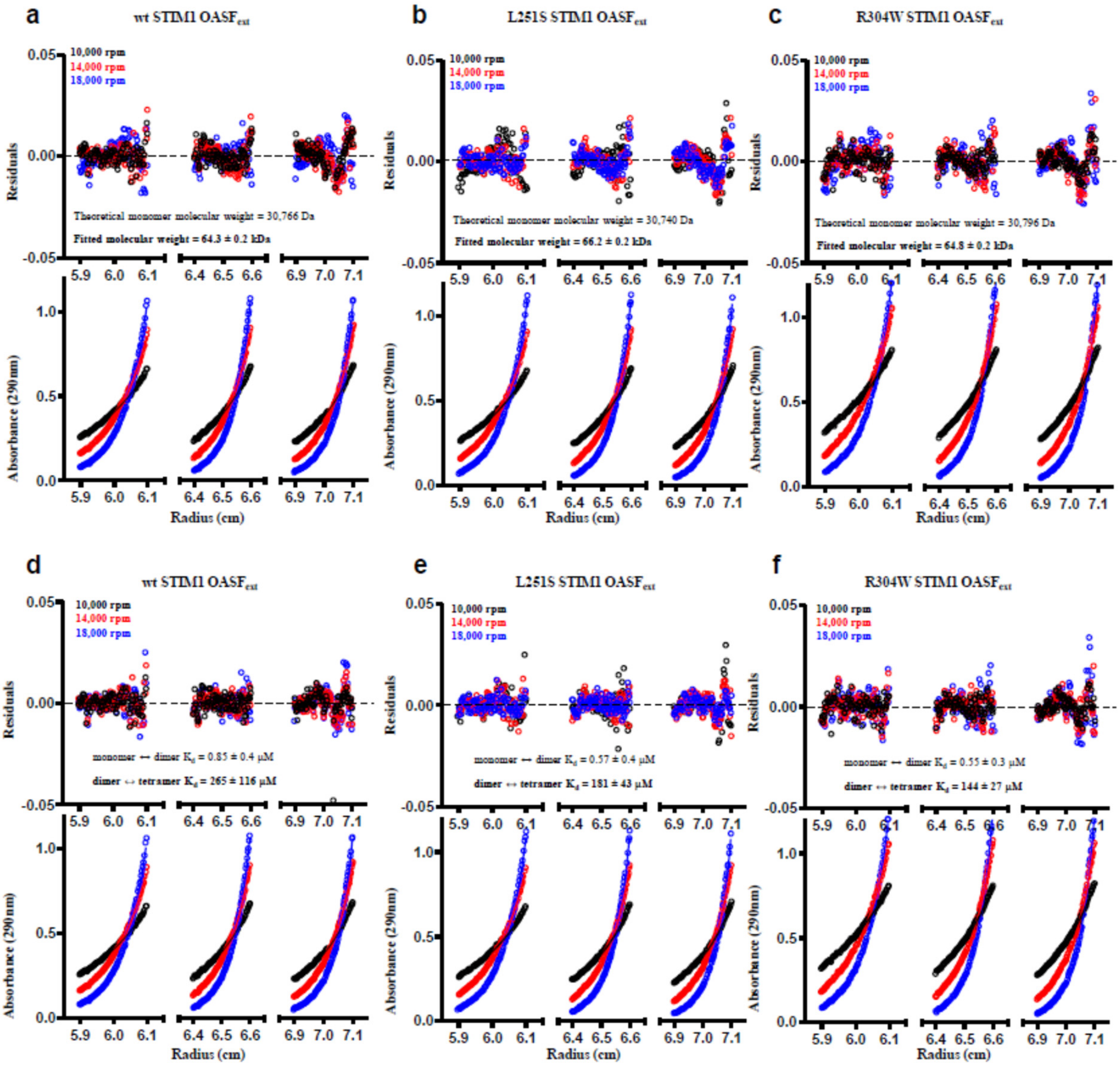

Supplementary Figure 3. Equilibrium ultracentrifugation analysis of STIM1 OASFext. (a) Global single ideal molecular weight fit to the sedimentation equilibrium data of wt STIM1 OASFext. The residuals of the global fit to the data are shown on the top of the panel. (b) Global single ideal molecular weight fit to the sedimentation equilibrium data of L251S STIM1 OASFext. The residuals of the global fit to the data are shown on the top of the panel. (c) Global single ideal molecular weight fit to the sedimentation equilibrium data of R304W STIM1 OASFext. The residuals of the global fit to the data are shown on the top of the panel. The fitted molecular weights correspond to 2.1, 2.2 and 2.1× the theoretical monomer weights for the wt, L251S and R304W proteins indicating that all proteins are primarily in the dimeric state under the conditions of the experiments. (d) Globally fitted equilibrium dissociation (Kd) of the dimer to tetramer association of the wt STIM1 OASFext. The residuals of the global fit to the data are shown on the top of the panel. (e) Globally fitted Kd of the dimer to tetramer association of L251S STIM1

OASFext. The residuals of the global fit to the data are shown on the top of the panel. (f) Globally fitted Kd of dimer to tetramer association of the R304W STIM1 OASFext. The residuals of the global fit to the data are shown on the top of the panel. In (e)-(f), the sub-mM tetramer Kd suggests a low self-association affinity for all proteins; however, consistent with the chemical crosslinking, the R304W protein shows a relatively higher affinity compared to wt. In (e)-(f), data were also globally fit to a monomer to dimer association model revealing sub-μM dimerization affinity for each protein (fitted curves not shown). In (*a*)-(*f*), the data were collected at 15 °C using 0.5 mg mL^{-1} of each protein in 20 mM Tris, 150 mM NaCl, 1 mM DTT, pH 8. Each panel shows 9 simultaneously fitted lines (solid lines through the data) corresponding to data of three replicate samples collected at three different centrifugation speeds (10,000, 14,000 and 18,000 rpm).

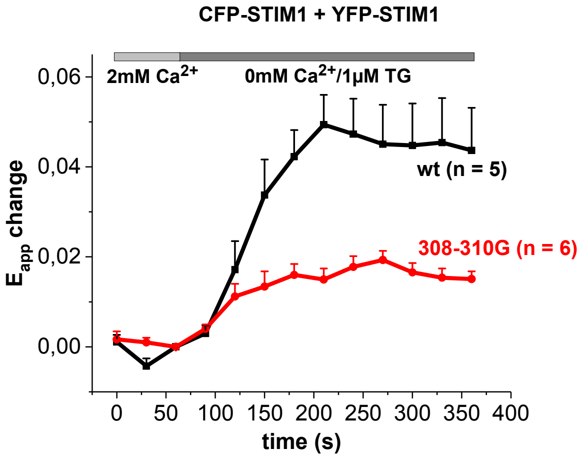

Supplementary Figure 4. FRET homomerization experiments of HEK293 cells co-expressing YFP- / CFP- STIM1 (308-310)G (red, n=6) or YFP- / CFP- STIM1 wt (black, n=5), respectively. Addition of 1 µM thapsigargin (TG) is used for store depletion. n-number in brackets. Error bars are defined as s.e.m.

Supplementary Table1

Primer Name	Primer Sequence (5' to 3')
R304Wfw	5'-CTCAGTACCCTCCCACAGCTCCTTCAGCC-3'
R304Wrev	5'-GGCTGAAGGAGCTGTGGGAGGGTACTGAG-3'
R304Ffw	5'-CTCATTCTCAGTACCCTCGAACAGCTCCTTCAGCCGCTG-3'
R304Frev	5'-CAGCGGCTGAAGGAGCTGTTCGAGGGTACTGAGAATGAG-3'
R304Lfw	5'-GCTGAAGGAGCTGCTGGAGGGTACTGAGA-3'
R304Lrev	5'-TCTCAGTACCCTCCAGCAGCTCCTTCAGC-3'
R304Vfw	5'-TTCTCAGTACCCTCCACCAGCTCCTTCAGCCG-3'
R304Vrev	5'-CGGCTGAAGGAGCTGGTGGAGGGTACTGAGAA-3'
R304Afw	5'-GGCTGAAGGAGCTGGCGGAGGGTACTGAGA-3'
R304Arev	5'-TCTCAGTACCCTCCGCCAGCTCCTTCAGCC-3'
R304Hfw	5'-CATTCTCAGTACCCTCATGCAGCTCCTTCAGCCG-3'
R304Hrev	5'-CGGCTGAAGGAGCTGCATGAGGGTACTGAGAATG-3'
R304Qfw	5'-TCTCAGTACCCTCCTGCAGCTCCTTCAGC-3'
R304Qrev	5'-GCTGAAGGAGCTGCAGGAGGGTACTGAGA-3'
R304Kfw	5'-ATTCTCAGTACCCTCCTTCAGCTCCTTCAGCCGC-3'
R304Krev	5'-GCGGCTGAAGGAGCTGAAGGAGGGTACTGAGAAT-3'
R304Efw	5'-CGGCTGAAGGAGCTGGAGGAGGGTACTGAGAA-3'
R304Erev	5'-TTCTCAGTACCCTCCTCCAGCTCCTTCAGCCG-3'
305-311Afw	5'-GCATATTTTTGGCGGCTCGCCGCAGCCGCAGCAGCCGCCCGCA-3'
305-311Arev	5'-TGCGGGCGGCTGCTGCGGCTGCGGCGAGCCGCCAAAAATATGC-3'
308-310Gfw	5'-GGCGGCTCCGCCCACCCCCAGTACCCTCC-3'
308-310Grev	5'-GGAGGGTACTGGGGGTGGGCGGAGCCGCC-3'
L300Wfw	5'-CCGCAGCTCCTTCCACCGCTGGGCTTCC-3'
L300Wrev	5'-GGAAGCCCAGCGGTGGAAGGAGCTGCGG-3'
L303Wfw	5'-CAGTACCCTCCCGCCACTCCTTCAGCCGCT-3'
L303Wrev	5'-AGCGGCTGAAGGAGTGGCGGGAGGGTACTG-3'
E305Wfw	5'-CTCATTCTCAGTACCCCACCGCAGCTCCTTCAGC-3'
E305Wrev	5'-GCTGAAGGAGCTGCGGTGGGGTACTGAGAATGAG-3'
E308Wfw	5'-CGGCTCCGCTCATTCCAAGTACCCTCCCGCAG-3'
E308Wrev	5'-CTGCGGGAGGGTACTTGGAATGAGCGGAGCCG-3'
Q314Kfw	5'-CTCCTCAGCATATTTTTTGCGGCTCCGCTCATTC-3'
Q314Krev	5'-GAATGAGCGGAGCCGCAAAAAATATGCTGAGGAG-3'

Q314Wfw	5'-AACTCCTCCTCAGCATATTTCCAGCGGCTCCGCTCATTCTCAG-3'
Q314Wrev	5'-CTGAGAATGAGCGGAGCCGCTGGAAATATGCTGAGGAGGAGTT-3'
Q314Afw	5'-CCTCCTCAGCATATTTTGCGCGGCTCCGCTCATTCT-3'
Q314Arev	5'-AGAATGAGCGGAGCCGCGCAAAATATGCTGAGGAGG-3'
E318Kfw	5'-CAACTCCTCCTTAGCATATTTTTGGCGGCTCCG-3'
E318Krev	5'-CGGAGCCGCCAAAAATATGCTAAGGAGGAGTTG-3'
E318Wfw	5'-CTCCAACTCCTCCCAAGCATATTTTTGGCGGCTCCGC-3'
E318Wrev	5'-GCGGAGCCGCCAAAAATATGCTTGGGAGGAGTTGGAG-3'
E318Afw	5'-CTGCTCCAACTCCTCCGCAGCATATTTTTGGCGG-3'
E318Arev	5'-CCGCCAAAAATATGCTGCGGAGGAGTTGGAGCAG-3'
L251Sfw	5'-AGGACTTGGAGGGGTCACACCGAGCTGAG-3'
L251Srev	5'-CTCAGCTCGGTGTGACCCCTCCAAGTCCT-3'
R426Lfw	5'-CATTGCGGGAGCTCCTGCACCGCTG-3'
R426Lrev	5'-CAGCGGTGCAGGAGCTCCCGCAATG-3'
431*fw	5'-GCCTGCACCGCTGGTAACAGATCGAGATC-3'
431*rev	5'-GATCTCGATCTGTTACCAGCGGTGCAGGC-3'
475*fw	5'-TGCTCACTTCATCATGACTTAAGACGTGGATGACATGGATG-3'
475*rev	5'-CATCCATGTCATCCACGTCTTAAGTCATGATGAAGTGAGCA-3'
EcoR1fw233	5’-TATATAGAATTCATGCAGAACCGTTACTCCAAGGAGCACATG-3’
EcoR1fw273	5'-TATATAGAATTCATGCGCACAGTGGAGGTGGAG-3'
EcoR1fw388	5'-TATATAGAATTCAACACACTCTTTGGCACCTTCCAC-3'
Sac2rev309*	5'-TATATACCGCGGCTAATTCTCAGTACCCTCCCGC-3'
Sac2rev342*	5'-TATATACCGCGGCTAATACCATGAGCTGTGAGATTCTAGCTCC-3’
Sac2rev474	5’-TATATACCGCGGAGTCATGATGAAGTGAGCAGG-3’

Supplementary Methods

OASFext expression and purification

STIM1 234-491 (i.e. OASFext) was expressed and purified using a pET-28a vector in BL21 DE3 *E. coli*. Briefly, the protein was extracted from the cells using 6M guanidine hydrochloride, isolated using Ni-NTA resin (Qiagen, Inc.) and refolded into 20 mM Tris, 300 mM NaCl, 1 mM DTT (pH 8.8). After thrombin digestion of the 6×His-tag, the protein was further purified by anion exchange chromatography using a HiTrap Q FF column (GE Healthcare) and finally exchanged into experimental buffer by dialysis. The R304W and R304A mutation, respectively, was introduced into the pET-28a OASFext construct using the QuikChange site-directed mutagenesis approach (Agilent, Inc.) and confirmed by DNA sequencing. Unless otherwise state, experimental buffer was 20 mM Tris, 250 mM NaCl, 1 mM DTT, pH 8.

SEC analysis

SEC was performed on an AKTA FPLC (GE Healthcare, Inc.) housed at 4 °C. A Superdex S200 10/300 GL column (GE Healthcare) was used at a flow rate 0.5 mL min^{-1}. One hundred twenty-five µL of protein at 0.75-1 mg mL^{-1} was injected, and the elution profile was monitored at a UV absorbance wavelength of 280 nm.

Glutaraldehyde crosslinking

Glutaraldehyde crosslinking was performed using 0.38 mg mL^{-1} of wt and R304W STIM1-234-491 protein solubilized in 20 mM Tris, 300 mM NaCl, 1 mM DTT, pH 8. Forty µL of protein was incubated at room temperature with 0 or 0.02 % (w/v) glutaraldehyde for 5 min. The crosslinking was stopped by icecold acetone protein precipitation. After pelleting of the protein by centrifugation, the protein was solubilized in 1× SDS PAGE loading dye, and samples were separated on a 10 % (w/v) SDS PAGE gel. Gels were stained using Coomassie blue dye and monomeric band intensities were evaluated using ImageJ software (v1.51h). The intensity of the monomeric bands of the crosslinked samples (0.02 % glutaraldehyde) were normalized to intensity of the monomeric band for the sample treated with no glutaraldehyde (0% glutarldehyde) [$I_{monomer(0\%)}/I_{monomer(0.02\%)}$]. Crosslinking was performed in triplicate for each experiment and repeated three times. Statistics are Student's t-test.

Far-UV CD spectroscopy

Far-UV CD data were collected using a Jasco-J-810 Spectrometer equipped with a PTC-423S temperature controller and a 0.1 cm pathlength quartz cuvette. Spectra acquisitions were made at 20 nm/min, 8 s response time, 1 nm data pitch with an average of 3 accumulations at 4 °C. Thermal

stabilities were acquired at 222 nm using a 0.1 cm pathlength quartz cuvette, 1 °C/min scan rate, 8 s response time, and 1 °C data pitch. CD spectra and thermal experiments were performed with protein concentrations of 0.2-0.5 mg mL^{-1} and 0.5 mg mgL^{-1}, respectively.

Analytical Ultracentrifugation

Sedimentation equilibrium studies were carried out using a Beckman Optima XL-A Analytical Ultracentrifuge. An An60Ti rotor and six-channel cells with Epon-charcoal centerpieces were used for the data acquisition. Absorbance measurements at 290 nm were collected in 0.002 cm radial steps and averaged over 10 readings. For stoichiometry calculations, the data from three replicate samples (for each protein) acquired at three centrifugation speeds (10,000, 14,000 and 18,000) were globally fit to a single ideal species model using a partial specific volume of 0.7335, 0.7326 and 0.7337 ml g^{-1} for the wt, L251S and R304W proteins, respectively. The buffer density used in the calculations was 1.012 g mL^{-1}. For monomer-to-dimer and dimer-to-tetramer Kd estimates, the molecular weights were fixed to the theoretical weights of the monomer and dimer, respectively, and a non-ideality term was included during the global fitting of the two-state equilibria to data from three replicate samples (for each protein) acquired at three centrifugation speeds.
